# Gender Differences in Public and Private Drinking Contexts: A Multi-Level GENACIS Analysis

**DOI:** 10.3390/ijerph7052136

**Published:** 2010-05-04

**Authors:** Jason C. Bond, Sarah C.M. Roberts, Thomas K. Greenfield, Rachael Korcha, Yu Ye, Madhabika B. Nayak

**Affiliations:** 1 Alcohol Research Group, 6475 Christie Avenue, Suite 400, Emeryville, CA 94608, USA; E-Mails: tgreenfield@arg.org (T.G.); scmr@berkeley.edu (S.R.); yye@arg.org (Y.Y.); mnayak@arg.org (M.N.); 2 NIAAA Training Program, School of Public Health, University of California Berkeley, Berkeley, CA 94709, USA; 3 Clinical Services Research Training Program, Department of Psychiatry, University of California, San Francisco, 401 Parnassus Avenue, San Francisco, CA 94143, USA

**Keywords:** context of drinking, on- and off-premises alcohol use, gender equity, economic development, culture, hierarchical linear models (HLM), cross-national study, GENACIS

## Abstract

This multi-national study hypothesized that higher levels of country-level gender equality would predict smaller differences in the frequency of women’s compared to men’s drinking in public (like bars and restaurants) settings and possibly private (home or party) settings. GENACIS project survey data with drinking contexts included 22 countries in Europe (8); the Americas (7); Asia (3); Australasia (2), and Africa (2), analyzed using hierarchical linear models (individuals nested within country). Age, gender and marital status were individual predictors; country-level gender equality as well as equality in economic participation, education, and political participation, and reproductive autonomy and context of violence against women measures were country-level variables. In separate models, more reproductive autonomy, economic participation, and educational attainment and less violence against women predicted smaller differences in drinking in public settings. Once controlling for country-level economic status, only equality in economic participation predicted the size of the gender difference. Most country-level variables did not explain the gender difference in frequency of drinking in private settings. Where gender equality predicted this difference, the direction of the findings was opposite from the direction in public settings, with more equality predicting a larger gender difference, although this relationship was no longer significant after controlling for country-level economic status. Findings suggest that country-level gender equality may influence gender differences in drinking. However, the effects of gender equality on drinking may depend on the specific alcohol measure, in this case drinking context, as well as on the aspect of gender equality considered. Similar studies that use only global measures of gender equality may miss key relationships. We consider potential implications for alcohol related consequences, policy and public health.

## 1. Introduction

There is increasing recognition that gender equality, along with other social factors, influence health [1,2] and thereby, public health. Many suggest that reducing gender inequality and patriarchy will improve health of both women and men [2,3]. However, it is possible that increases in gender equality may lead to women adopting riskier and traditionally more male health behaviors, including smoking and alcohol consumption. These health behaviors, in turn, may negatively impact health outcomes. For example, one study found that the association between macro-level gender equality and women’s mortality was partially mediated by changes in smoking [4].

The relationships between macro-level gender equality and different health outcomes, including morbidity, mortality, reproductive health, mental health, tobacco, and violence (all of public health significance), have been explored [5–10]. A number of recent studies have sought to document convergence (or a reduction in the size of gender differences) in alcohol patterns and consequences [11–14]. However, only one published paper examined the relationship between macro-level gender equality and alcohol consumption and consequences [15]. This study generally found that increased gender equality predicted a convergence in alcohol consumption and consequences.

Despite a lack of research, some scholars attribute convergence in alcohol consumption to increased gender equality (see, [14] quoted in [16]). In addition, articles in the popular press have focused on gender convergence in drinking and increases in women’s drinking and cite drunk women and women drinking in public settings, such as bars, as examples of the failures of feminism and the downside to increased gender equality [16–18]. Despite the widespread popularity of this concern about increases in women’s drinking, especially in public settings, and the associated attribution to gender equality, only a few studies have documented gender differences in drinking in public settings such as bars and restaurants or drinking in private settings such as homes [19–22]. No research has explored the relationship between macro-level gender equality and the size of gender differences in drinking in each of these settings in a comparative multinational framework.

From a public health perspective, drinking in public settings, especially bars, may be a key alcohol behavior to monitor and understand since drinking in public settings is often associated with specific negative consequences for both males and females [23]. In U.S.-based studies, drinking in public, on-premise locations such as bars and taverns is associated with heavier drinking patterns [24–26]. It has been proposed that such settings provide cues and social learning mechanisms that reinforce heavy drinking [22,27]. In addition, a recent study found that consuming the largest amount of alcohol in bar settings compared to home was associated with increased alcohol-related consequences, controlling for overall alcohol volume and frequency [22]. In North America, for example, drinking in bars and particularly certain types of bars has often been found to be associated with elevated risks of alcohol problems including aggression [28,29], sexual risk taking [30], other drug use [31] and most especially drunk driving [32]. Bar patronage may be associated with problems in other countries too (e.g., South Africa [33]). Generalizations from the individual to the ecological level are subject to the atomistic fallacy [34]. However, the consistency of findings regarding increased levels of drinking and harms associated with drinking in public settings suggests that characterizing countries based on the level of drinking in public settings may be important in understanding geographic variation in alcohol-related behavioral risks. Here, we focus on frequency of drinking by venue type; exploring the relationship between level of drinking in public settings and harms is beyond the scope of this analysis. However, because of the relationship between drinking level in different settings and harms for men and women, while an important topic [22], we briefly review the public health rationale for examining gender in relation to drinking context, considering how this might affect harms.

The size of the gender difference in drinking in public settings is not solely a popular concern. It is plausible that the gender difference in drinking in public settings may play a role in determining alcohol-related consequences associated with drinking in that setting. The size of gender differences in drinking in public settings can be seen as an indicator of the “genderedness” of the drinking context. The “genderedness” of a drinking context could, in turn, influence consequences associated with drinking in that context either by influencing who chooses to drink in that context or by changing the way the context influences the drinker. For example, women drinking outside the home in settings where doing so is (or has been) a mostly male activity has been seen as a marker of gender deviance [35–38]. Women who drink in bars may be perceived as sexually promiscuous and inviting sex and sexual assault [35,37,39,40]. Also, mostly (or all) male and therefore “masculine” drinking contexts may also contribute to both heavy consumption patterns and certain consequences, as alcohol consumption in public contexts is one way through which men construct masculinity, or “be men” [41–43]. Thus, both men and women who tend to drink more often in bars in countries where there is a large gender difference in drinking in public settings may be at greater risk for harms. Harms for women could plausibly increase or decrease as the gender difference in drinking in public settings decreases.

The growing literature on effects of macro-level gender equality on health [5–10] offers some guidance for study design and for measuring macro-level gender equality. While longitudinal studies would be the preferred study design, lack of data makes such studies difficult. As an alternative strategy, many studies in the larger literature on gender equality and health look at variation across geographic locations, such as countries, states, and cities [4,5,7].

In addition to using composite indicators to measure overall gender equality, the literature on gender equality and health generally measures and explores the following domains: gender equality in economic participation and opportunity, gender equality in education, gender equality in political participation, control of reproduction, and context of violence against women [44]. Gendered labor and gendered power [45] may be especially relevant for understanding gender differences in drinking in public, and possibly private, settings. In relation to gendered labor, performance of and gender role expectations relating to daily tasks, such as employment outside the home, parenting, and housekeeping may both vary across countries and influence alcohol use [46–48]. Group- or country-level variation in gender role expectations relating to which daily tasks, such as employment outside the home, parenting, and housekeeping, women and men are expected to perform may also influence gender differences in drinking. In countries where there is more gender equality in economic participation and opportunity, we would expect smaller gender differences in drinking, especially in drinking in public settings. In relation to gendered power, widespread acceptance of beliefs about negative meanings of female drinking in bars and the resulting threat of sexual assault may, as suggested by feminist theory [49], lead fewer women to consume alcohol in public settings, especially bars. Thus, in countries with high levels of violence against women and little state response to such violence, we would expect greater gender differences in drinking, especially in public settings.

This study takes the first step in building a research program based in the area of gender differences in drinking in different settings, gender equality, and, to be studied later, alcohol related-harms. This study first characterizes twenty-two countries from the developed and developing world by the size of gender differences in frequency of drinking in public and in private settings. It then explores whether country-level gender equality modifies the size of gender differences in frequency of drinking in two major settings- on premise (bars and restaurants) and off premise (e.g., homes), controlling for country-level economic status and individual-level factors. Because indicators of country-level gender equality mostly measure equality in the public sphere, we hypothesize that indicators of macro-level gender equality will be more likely to predict the size of gender differences in drinking in public than in private settings. While plausibly connected to alcohol related harms, exploring the connections between gender differences in drinking in public and private settings and harms is, as noted, beyond the scope of this initial analysis.

## 2. Methods

### 2.1. Data Sources

Survey samples come from the multi-country GENACIS project [50]. Twenty-two of the GENACIS countries were included in this study. These countries are at varying stages of development and in several geographic areas across six continents [See Table 1]. Methods were mostly similar across countries, although there was variation. See Table 1 for details. Surveys in each country were conducted between 2000 and 2007. Many sampling frames were national or nearly national, whereas others represented a state (e.g., in India) or areas within a country. Regional studies generally focused on large populations centers within the country. In several cases, the areas within the country account for more than 50% of the country’s total population.

Some surveys were conducted face-to-face by trained interviewers; others involved telephone surveys or combined telephone and postal surveys. In some cases, sampling used random digit dialing techniques or was register based. In many cases, multi-stage cluster sampling was used, stratifying by district or some other regional descriptor. In the majority of cases, one individual in the age range (typically over 18, but sometimes with an upper age cap of 65 or 75) was randomly or systematically selected per enumerated or selected household. The average sample size per country was 1,270 men and 1,054 women. Per the GENACIS study objectives, nearly all datasets, with the exception of Brazil and Isle of Man, include a minimum sample size of 1,000. The datasets from the United States and Canada were substantially larger. Because of gender differences in abstention, actual *n*s of male and, especially female, current drinkers vary greatly and are small in some cases. Although women’s full samples were adequate (Table 1), there are small numbers (under 100) of current female drinkers in Brazil, India, and Sri Lanka. Response rates ranged from 38%–96% with a median of 64% with further details of the sampling design across countries available in [51].

### 2.2. Measures

#### 2.2.1. Dependent variables

This study examines two separate dependent variables: frequency of drinking in public settings and in private settings over the past 12 months. Frequency, rather than usual quantity or volume, was used because only frequency and not quantity in different settings was collected in the surveys. These variables are based on the GENACIS Expanded Core questions. The surveys assessed frequency of drinking in various contexts by asking: “Thinking back over the *last 12 months,* about how often did you drink in the following circumstances? *Think of all the times that apply in each situation”.* Two situations, or contexts, were chosen to represent Public drinking: “in a bar/pub/disco” and “in a restaurant” and two were chosen to represent Private drinking: “at a party or celebration” and “in your own home”. The eight response categories ranged from “every day or nearly every day” through “once or twice a year” to “never in the last 12 months”. Categories were converted to the metric of days per year using category midpoints. The values for each of the two constituent contexts were summed to indicate the frequency of drinking in each (public and private) setting. Because it is possible to drink in two settings on a given day, the summed frequencies could exceed 365 days. However, exceeding 365 days was extremely rare, so results were not truncated.

Identical or similar questions were asked in each participating country. Sweden only asked these questions in a random third of the full sample; however, the one-third sample was similar in size to those of other countries (Table 1). Most countries included the two separate questions for frequency of drinking in public settings, *i.e.*, in (a) a bar, pub, or disco and (b) restaurant. However, Denmark, Iceland, and Sri Lanka surveys asked about frequency of drinking in a bar, pub, disco, or restaurant/café in a single combined question. Asking multiple questions tends to give higher values than use of a single, combined question. To make responses from surveys more comparable and reduce the methodological ‘penalty’ in the three surveys with the single public setting question, gender specific ratios of frequency of drinking in bars, pubs, and discos *versus* in restaurants from similar countries were applied to the gender-specific combined public venue data. For Denmark and Iceland, Swedish ratios were applied. For Sri Lanka, Indian ratios were applied. Restaurant drinking was minimal in India and in Sweden, so this adjustment made very little practical difference.

#### 2.2.2. Independent variables

Country-level variables: Country-level variables to measure gender equality and economic status include both existing indices and indices created specifically for this study.

***Gender equality*** We included four existing indices and two newly created indices to measure composite gender equality; gender equality in economic participation and opportunity, education, and political participation; reproductive autonomy; and context of violence against women. The existing indices were: the 2008 Gender Empowerment Measure (GEM) and the 2007 Global Gender Gap Index (GGI) Economic Participation and Opportunity, GGI Education, and GGI Political Participation sub-indices [52–54]. Indices of women’s reproductive autonomy and context of violence against women were created. In addition to the theoretical reasons for including gender equality in economic participation and opportunity and the context of violence against women described in the introduction, the GEM and other indices were included mainly because of their use in previous research related to gender equality and health [7–9,15,55].

The GEM is a composite index that measures gender equality in political participation and decision-making, economic participation and decision-making, and power over economic resources. Higher scores indicate greater gender equality. Sweden has the highest GEM score (0.925), with Denmark and Iceland also highly ranked (0.887 and 0.881 respectively). India, Sri Lanka, and Nigeria have low GEM scores (0.24, 0.371, and 0.198 respectively); while Costa Rica, Argentina, and the United States have moderate scores (0.69, 0.692, and 0.769 respectively). GGI sub-indices estimate relative to men, women’s economic participation and opportunity, educational attainment, and political participation. Higher GGI scores indicate greater gender equality The GGI also has a composite index of country-level gender equality. However, the GGI composite index includes gender differences in life expectancy. Therefore, the GEM was preferred as the composite gender equality indicator.

To our knowledge, there are no existing indices that measure women’s reproductive autonomy across countries. However data about reproductive autonomy and women’s actual control over reproduction are consistently collected and reported in multiple sources. We created a reproductive autonomy index based on the following variables: restrictiveness of abortion laws [56], contraceptive prevalence [57], total fertility rate per woman [58], mean age at marriage for women [57], and length of maternity leave [57]. This index reflects a combination of both policy-level reproductive rights and actual reproductive control by women. Adolescent fertility rate and modern contraceptive use were also considered. They were not included because of high correlations with the previously mentioned variables and more missing values. Country-specific indices were created through factor analysis of the five variables: restrictiveness of abortion laws (a five category variable with 1 being most restrictive, 5 least), prevalence of any contraceptive use, total fertility rate per woman, mean age at marriage, and average number of weeks available for maternity leave. A factor analysis revealed a strong single dimensional structure (first eigenvalue of 3.1 comprising 63% of the total variance, all factor loadings larger than 0.7, and with the second eigenvalue less than 1).

To our knowledge, there are also no existing indices that measure the context of violence against women across countries. Recently, many countries have started to collect data on both actual violence against women and countries’ responses to this violence. However, data on actual rates of violence against women are collected inconsistently and are often not comparable across countries. Recent attempts to standardize data collection have moved in a positive direction (see, for example [59]). However, such data is available from only a subset of countries. Because context of violence against women is a theoretically important factor for this analysis, we created an index based on the best available data. This includes both actual rates of violence against women and country response to such violence. The index is based on the following variables: percent ever sexually assaulted (either attempted or completed) [60–64], percent experiencing physical violence by a partner in the past year [59,61,62,65], percent of population feeling unsafe on the street after dark [66,67], homicide rates [61,67–69], attitudes towards wife beating [59,60,70–72], quality of violence against women legislation [73], and the number of domains of activity to address violence against women a country engages in as reported to the UN Secretary General [74]. Even with use of multiple sources, many values of these variables were missing.

Country-specific indices were created through factor analysis. Variables included in the factor analysis were chosen based on completeness of data and findings from pairwise correlations with a wider range of measures that included varying time frames for sexual assault and partner physical violence as well as gender-disaggregated homicide rates. Only total homicide rate was included because there were more missing data for gender disaggregated rates and male and female homicide rates were highly correlated (0.75). The seven variables included: percent of women reporting ever being sexually assaulted, percent of women reporting physical violence against them by a partner in the past year, percent of the population feeling unsafe on the street after dark, rate per 100,000 of mortality caused by homicide, percent of men reporting that violence towards one’s wife was justifiable, quality of legislation within the country punishing violence against women (on a scale of 0–1 with 0 as the highest quality of legislation and 1 as the lowest quality of legislation), and the number of different domains of activity to address violence against women that a country engages in, as reported to the UN Secretary General (on a scale of 0–1, with 0 as having activities in all 7 possible domains). The percent of the population feeling unsafe on the street after dark was initially included in the factor analysis, but produced a very small factor loading and was therefore excluded. Similar to the results for the reproductive autonomy factor, a strong single dimension emerged from the analysis (first eigenvalue of 3.7 comprising 62% of the variance, all factor loadings larger than .6, and with a second eigenvalue of less than 1).

***Economic status*** Gross Domestic Product per capita 2006 (GDP) and the Human Development Index (HDI) were both considered as indicators of country-level economic status. The two measures were highly correlated (0.82) and multilevel findings from the HDI were similar to GDP. Therefore, only results for GDP are reported here as it was less correlated with the other country level variables than the HDI (See Table 3). Differences in findings between HDI and GDP are noted in the text.

***Missing values*** Data for each country, with the exception of Isle of Man, were available for GGI sub-indices and GDP. Missing data was dealt with by substituting values with those from similar countries and by multiple imputation. First, country-level data for the Isle of Man were unavailable. Data from the United Kingdom was deemed to be the most appropriate country based on both current and prior British influence and therefore were directly substituted. Second, for the 2008 GEM data were unavailable for India and Nigeria. GEM scores from 1999 and 1996, respectively, were used.

As standard HLM models require complete country-level data and as the pattern of missingness of country-level data was well-dispersed across countries, the remaining missing values were imputed within each gender equality domain. For the reproductive rights index, Iceland was missing the contraceptive prevalence rate. For the context of violence against women index, values for 24 country-variable pairs (about 22% of observations) were missing. The missing values were for sexual assault (eight countries), physical violence from partner (seven countries), and quality of legislation punishing violence against women (one country). Data were imputed in the NORM program [75]. This program imputes continuous data assuming a multivariate normal distribution of the data. Missing data were imputed 10 times and the average value was substituted for all missing values. In order to examine the variability in the estimates produced from the multiple imputations, the range of each of the imputed values was estimated. For all data imputed, variation across imputations was very small. The range from the smallest to the largest values across all variables ranged from only −1% to +2%. Given the small amount of variability in imputations, it was decided that it was unnecessary to estimate the model for each of the multiply imputed datasets and then combine resulting model estimates.

Individual Level Variables: Age, gender, and marital status were taken from responses to the GENACIS surveys in each country. Across countries, age was asked as a continuous variable. Marital status, although asked with slightly different possible categories across country, was coded as 1 if the respondent was married or living with a partner and 0 otherwise. Gender was coded as 0 if female and 1 if male.

### 2.3. Analysis

Hierarchical Linear Modeling (HLM) [76], using HLM V6.08 [77], was used to study variation across countries in gender differences in frequency of drinking in public and in private settings and to determine whether country-level gender equality modifies the relationship between women’s and men’s drinking in each setting. Frequencies are required to be whole numbers zero or greater and the distribution of the raw frequencies was somewhat skewed to the right in each country. Thus, the natural log transformation (ln of 1+ the frequency, to avoid problems with zeros) was used.

Marital status and age were each centered around their overall means in order to obtain interpretable intercept and gender coefficients from the HLM model. Each country level variable was centered and scaled to have a mean 0 and a variance 1, for ease of comparison. Sampling weights, accounting for survey design, were used for all analyses.

Separate models were estimated for drinking in public and in private settings and for each country-level gender equality and economic status predictor. The model for drinking in public settings using the reproductive rights measure is presented here for illustrative purposes. The model estimated was:

$$\begin{array}{c} \text{ln}(1+y_{i,c})=\alpha_{c}+\beta_{c}G_{i,c}+\lambda_{1}M_{i,c}+\lambda_{2}A_{i,c}+{ɛ}_{i,c}\\ \alpha_{c}=\gamma_{0,0}+\gamma_{0,1}Z_{c}+u_{0,c},\beta_{c}=\gamma_{1,0}+\gamma_{1,1}Z_{c}+u_{1,c} \end{array}$$

where *y_i,c_* is the frequency of drinking in public settings for the *i^th^* respondent in the *c^th^* country, *A_i,c_* their age, *M_i,c_* is their marital status, and *G_i,c_* is an indicator for whether the respondent was male. The variable Z*_c_* is the country-level predictor (here, reproductive control). This two-level model contains two random effects: a random intercept and a random gender coefficient. Individual-level marital status and age were assumed to be fixed effects. The random effects u*_0,c_* and u*_1,c_* were assumed to be distributed normally with variance-covariance matrix T and were assumed to be independent of the normally distributed country level variance term ɛ*_i,c_* which was assumed to be independent across individuals. Therefore, the interpretation of the intercept α*_c_* is that of the country-specific frequency of drinking in public settings for women at the average age and for the average proportion married. The interpretation of the random gender coefficient β*_c_* is the country-specific difference in frequency of drinking in public settings between men and women at the average age and for the average proportion married. A significant positive coefficient for *γ_0,1_* would suggest that, as women in a country gain more reproductive autonomy, women’s frequency of drinking in public places increases. Similarly, a significant positive coefficient for *γ_1,1_* would suggest that increased rights would be associated with a smaller difference in the frequency of drinking in public settings between men and women.

In addition to separate models for each country-level predictor for both public and private settings (models 2), a final model (model 3) was estimated to examine which, if any, of the country-level equality indicators were still significant after controlling for GDP. This model used a forward stepwise procedure where all country-level variables that were significant in the separate models were entered simultaneously, starting first by forcing the entry of GDP into the model. The condition number for the correlation matrix (Table 3) of the country-level variables—ratio of largest to smallest eigenvalue was 207 with a smallest eigenvalue of 0.03—indicated an acceptable level of correlation for inclusion of multiple covariates in the multivariate model. In order to have greatest relevance to public health, we emphasize population-based results. Separate models were also estimated for drinkers only, and where results differ, are described in the text.

## 3. Results

### 3.1. Descriptive Results

The samples sizes, proportion of the population drinking within the past 12 months, and frequency of drinking in public and, in private settings, are shown in Table 2. Rates of current drinking varied considerably across the countries for which data was available. There was more variability across countries for women than men. For women, current drinking rates varied from a low of 3.0% in India and 5.8% in Sri Lanka to 93.8% in Denmark, with an average of 59%. For men, drinking rates varied from a low of 36.9% in India to 96.8% in Denmark with an average of 72.6%.

**Figure 1.**
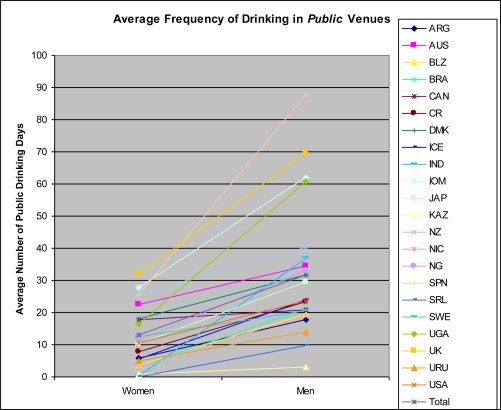
Estimates of Frequency of Drinking in *Public* Venues for the Full Sample.

**Figure 2.**
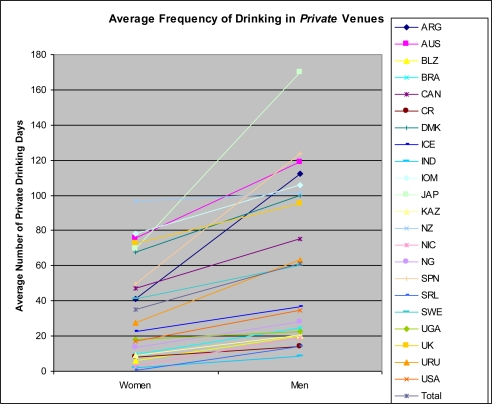
Estimates of Frequency of Drinking in *Private* Venues for the Full Sample.

Table 2 and Figures 1 and 2 show the mean frequency of drinking in public and private settings for both women and men and gender differences in drinking in each setting for the entire sample. There appears to be variation due to gender, setting, and non-gender specific frequency of drinking. First, women consistently drink less frequently than men in both public and private settings. Second, in the majority of countries, both women and men drink more frequently in private than in public settings. The latter differences are small, and in some cases reversed, in about one fourth of the countries, mostly in Latin America, Africa, and South Asia. Third, mean frequencies of women’s and men’s drinking are highly correlated in both public (0.95) and private (0.94) settings. But, the size of the gender difference in mean frequency of drinking in public settings is not correlated with that of private settings (0.16).

The relative frequency of drinking in each setting across countries varies less for women than for men. For women, the order of countries from least to greatest mean frequencies is roughly similar across public and private settings. For men, the order of countries is more heterogenous across the two settings. This difference can be seen by ranking each of the public and private frequencies separately and then summing the corresponding paired differences of these ranks as well as by Pearson correlations between mean frequency of drinking in each setting. For women, the summed difference is 52 and the correlation is 0.92. For men, the summed difference is 154 and the correlation is 0.04.

The size of the gender difference in drinking appears influenced by both men’s and women’s drinking, but does not appear to be due to overall frequency of drinking in the country. Specifically, higher mean frequencies of drinking for women and lower mean frequencies of drinking for men were associated with smaller expected gender differences in each setting, although these associations were stronger for women’s than men’s frequency. For women, correlations between the intercept and gender slope coefficients for Model 1 (Table 4) were moderate and negative, taking values of −0.25 and −0.65 for public and private drinking, respectively. For men, these correlations were 0.07 and 0.26 respectively. Large gender differences in drinking in public settings exist both in countries where women drink infrequently (such as India, Belize, Nicaragua, and Sri Lanka) and in countries where women drink relatively frequently in public settings (such as Spain and the United Kingdom).

### 3.2. Multi-Level Modeling Results

Values for each country-level variable are available from the first author. Table 3 provides Pearson correlation coefficients among these variables.

Estimates for the multilevel models can be seen in Table 4. Model 1 shows coefficients for the HLM model that includes only individual-level variables along with random effects for the intercept and the gender coefficient, but does not include any country-level variables. Model 2 includes coefficients for each country-level variable entered in separate models. Model 3 includes coefficients for country-level gender equality variables entered separately into a model where GDP has already been included (only variables with significant coefficients are shown).

#### 3.2.1. Public setting results

Married people report significantly lower frequency of drinking in public settings than unmarried people (See Table 4, Model 1). In addition, frequency of drinking declined with age and men reported a significantly higher mean frequency of public drinking than women across all countries.

The within country gender differences in mean frequencies are indicated by the slopes of the lines connecting the paired estimates in Figure 1 and are characterized by the gender coefficient estimate. Country-level coefficients for Model 2 are shown in Table 4 for only the gender slope coefficient (*i.e.*, the gender difference) and not for the gender intercept. All country-level gender equality and economic status indicators, with the exception of gender equality in political participation, predicted the gender slope coefficient. For each significant association, increased gender equality and economic status predicted a smaller gender difference in frequency of drinking in public settings. Context of violence was reverse coded, which is the reason the sign of the coefficient is positive. Because country level variables were standardized, the magnitude of the coefficients can be compared. Most coefficients were near 0.15. The coefficient for economic participation and opportunity was somewhat larger (0.21) and that for gender equality in educational attainment was somewhat smaller (0.11).

Analyses in Model 2 were also conducted for current drinkers only (results not shown). While gender slope coefficients were slightly larger in magnitude on average (ranging from 0.2 to 0.3), each country-level indicator that was significant in the whole sample was again significant at similar levels of significance as for the entire sample. Political participation was again not significant. There was also very little change in individual-level coefficients once country-level variables were entered in Model 2.

The stepwise forward entry model results where GDP was forced in and essentially controlled for (Model 3) are shown in Table 4. For drinking in public venues, after forcing GDP, gender equality in economic participation and opportunity entered the model as the most significant second predictor; GDP was reduced to non-significance. No other country-level variable other than gender equality in economic participation and opportunity was significant after controlling for GDP.

#### 3.2.2. Private Setting Results

Unlike results for public settings, married people reported significantly higher frequency of drinking in private settings than unmarried people and age was not associated with frequency of drinking (See Model 1, Table 4, rightmost column). Also unlike the public setting results in Model 2, only gender equality in educational attainment was significantly associated with the gender slope coefficient. A higher level of equality in educational attainment was associated with a larger difference (*p* < 0.05) between men’s and women’s frequency of drinking in private settings. However, after entering both GDP and equality of educational attainment (Model 3), educational attainment was no longer significant (*p* = 0.16).

For the sample of current drinkers only (results not shown), more gender equality in educational attainment (*p* = 0.003), less violence against women and more state response to the violence (*p* = 0.02) and higher values of the HDI (*p* < 0.001) were each associated with larger gender differences in frequency of drinking in private settings. Multivariate models were not estimated.

## 4. Discussion

As has been found in previous research about gender differences in drinking more generally [78], we found that men consistently drink more than women in each setting. While variation in both women’s and men’s drinking appear to contribute to the size of the gender differences in drinking, the size of the gender difference is more highly correlated with frequency of women’s than men’s drinking. This is more the case in private than public settings. However, non-gendered factors also seem to play a role as women’s and men’s average frequencies of drinking in each setting across countries are highly correlated. Factors that influence frequency of drinking do not have an obvious relationship with the size of the gender difference since large gender differences in drinking in public settings occur both in countries where women drink infrequently and countries where women drink frequently. Finally, and a key finding for research on contexts of drinking, the size of the gender difference in drinking in public settings is not correlated with the gender difference in drinking in private settings. This suggests that drinking in public and drinking in private settings are distinct drinking behaviors and deserve separate consideration in studies of gender and alcohol. From a public health perspective, the distinction is also important since many problems are more associated with drinking more in public rather than in private settings [22].

This is the second cross-national study to examine the relationship between gender equality and gender differences in alcohol consumption and the first to examine the relationship between gender equality and drinking in specific settings. Findings are consistent with the theory that higher country-level gender equality, especially in economic participation and opportunity, predicts smaller gender differences in frequency of drinking in public settings. In the case of certain gender equality variables, this relationship persists even after controlling for country-level economic status. These findings support the hypothesis that it is gendered labor, or gender differences in work and material resources, that most influence gender differences in drinking in public settings. They do not support the hypothesis that gendered power, as operationalized by context of violence against women, predicts the size of the gender differences. They also suggest that gender equality in education and reproductive autonomy may be relatively less important and political participation unimportant for studying gender equality and alcohol use in public venues.

Most country-level variables did not explain the gender difference in frequency of drinking in private settings. Where gender equality predicted this difference (educational attainment), the direction of the findings was opposite from the direction in public settings, with more equality predicting a larger gender difference. However, this relationship was no longer significant after controlling for country-level economic status.

That smaller gender differences in frequency of drinking in public settings are associated with both higher frequency by women and lower frequency by men suggest two competing plausible implications that need to be researched further. Hypothetically, smaller gender differences could imply that increased equality in economic participation will lead women to face increased alcohol-related consequences, including but not limited to sexual assault. Conversely, an alternative possibility is that smaller gender differences in public drinking might lead these contexts to be less “masculine,” and so could reduce the well-documented harms [22,24–26,28–33] associated with drinking in these settings by lessening the incentives to drink and fight so as to demonstrate masculinity. More research is needed to test these hypotheses, which are beyond the scope of present analyses focused only on frequency of drinking by men and women in these settings.

### 4.1. Limitations

This study should be interpreted in light of its limitations. First, as this is a cross-sectional study, it is not possible to determine if increases in gender equality caused smaller gender differences. It is possible that in countries where both men and women drink frequently in public settings, barriers to women’s economic participation will be smaller. Longitudinal data is needed to determine the direction of this relationship. Second, country-level variables are derived from multiple data sources. Although attempts were made to match years of country-level variables and survey years, country-level data do not correspond exactly to survey year in each instance. Third, the indicator of context of violence against women was assembled from multiple sources and data availability required the use of different time frames and definitions for prevalence of sexual and physical violence. It includes both violence against women and overarching violence (*i.e.*, homicide rates). Despite these limitations, most of the variables loaded on one factor, suggesting that it was meaningful to include this theoretically important factor in our analysis. Still, coefficients for the context of violence against women index should be interpreted with some caution. Fourth, regarding the measures of drinking in public and in private settings, the private measure may be imperfect, as private includes drinking at parties, which could be considered to be either a public or a private setting. However, we considered primarily the on- *versus* off-premise (on-sales *vs.* off-sales) notion, important from a policy perspective, since parties are often ‘closed’ and thus formally private, and generally cannot be regulated as can bars and restaurants which are, in many cases, licensed venues. Fifth, for this secondary analysis, only frequency of drinking in each setting, and not volume or usual quantity consumed in that setting, was available due to limitations in most of the GENACIS surveys. If alcohol-related problems are, as seems likely, more a function of volume or quantity consumed, then only to the extent that frequency is correlated with these will the results be relevant to alcohol-related harms. Previous research has found that frequency of drinking in bars, and not just intoxication in bars, has been linked to increased risk for victimization for women [79]. Additionally, some previous research has found that frequency of drinking may predict more alcohol-related problems than binge drinking at the same or lower volumes [80]. Additional analysis of GENACIS data suggests that although overall drinking frequency is not as strong a predictor of harms as usual quantity, frequency of binge drinking, or volume (with standardized effect size is about half that of these other 3 consumption measures) alone it remains a strong and significant predictor or harms in and of itself. Sixth, some of the correlations among country-level variables, especially GDP and the other gender equity variables considered may be considered high. It may be that this shared variability is that which is most associated with variability in the outcome, therefore making it difficult to remove the effects of confounding of GDP on the GEM, education, reproductive autonomy, and context of violence against women and gender differences in public drinking associations. Last, the set of countries available, though diverse, represents a kind of convenience sample of countries. In addition, the effect of the diverse range of survey methodologies and response rates on the results here are not known. The range of countries included, however, is broader than many cross-national studies to date, none of which have either documented or sought to explain gender differences in drinking in different settings.

### 4.2. Strengths

This study also has a number of strengths. First, it tests theory—specifically that macro-level gender equality will influence gender differences in drinking in public settings and will less so or not at all influence drinking in private settings. Findings are consistent with part of this theory, mainly that gender equality in economic participation predicts size of gender differences in drinking in public settings, thus helping support this conceptualization. Contributions include strengthening the position that (1) macro-level gender equality may influence public more than private behavior; (2) the relationship between macro-level factors and alcohol consumption may differ for drinking in different settings; in turn suggesting that future research should account for drinking context; and (3) identification of indicators of macro-level gender equality, mainly gender equality in economic participation and opportunity, may be important for alcohol research and investigation of alcohol-related outcomes in cross-national studies. Second, the sample (22 countries) included countries with comparable drinking context questions based on a core questionnaire developed by an international group of scholars and generally comparable methods [51], thus making cross-country comparison possible. The GENACIS data represents a unique opportunity for investigating hypotheses cross-nationally and shaping hypotheses for testing in later studies.

## 5. Conclusions

Macro-level gender equality may influence alcohol consumption patterns (here, drinking in public settings). Consumption patterns, including the preferred environments in which one drinks, in turn are known to influence alcohol-related harms [22]. The effects of gender equality on drinking may depend on the specific alcohol measure, in this case drinking context, as well as on the aspect of gender equality considered. In this case, gender equality in economic participation and drinking in public settings appear to be important alcohol and gender equality measures to study further. Studies that including only global measures of gender equality such as the GEM and do not consider drinking context may obscure key relationships between gender equality and alcohol use. Our findings that gender equality in political participation was not associated with gender differences in drinking in either setting calls into question the utility for alcohol research of composite indicators such as the GEM, which include political participation variables. We believe relationships between gender equality and alcohol has implications for alcohol-related harms as well as health more generally. Future research is needed to determine which aspects of gender equality matter for which alcohol-related behaviors. Specifically, related to the findings from this study, research is needed to explore the relationship between gender equality and other alcohol behaviors as well as to determine whether and how changes in genderedness of settings in which alcohol is consumed lead to more or less harm for women and men. To further the public health agenda, we need additional research with larger samples of countries, better measures of some indicators of gender equality, mainly context of violence, gender-disaggregated drinking patterns (rather than only gender differences), and indicators of alcohol-related harms to further understand these relationships.

### 5.1. Implications

From a public health perspective, the findings from this study offer some suggestions for strategies to prevent alcohol related harms and also raise important questions for future research to inform such strategies. First, the characterization of countries in this study by frequency of drinking for both women and men provides information for locating interventions. For example, in countries where women drink frequently in public settings, it is appropriate to locate alcohol interventions, such as responsible beverage service programs, in public settings. Second, this characterization provides information for the content of alcohol-related interventions. Countries where the majority of drinking by men takes place in public settings may benefit from increased regulation of on-premise drinking while countries where the majority of drinking by men takes place in private settings may require a focus on regulating off-premise liquor sales. Research is needed to understand the different types of harms for both men and women associated with drinking in settings with large and small gender differences and whether there is a tipping point at which the level or type of harms begins to change. Both qualitative and quantitative studies will be needed. Such research will inform the content of future interventions. Finally, it is also not yet clear how changes in frequency of drinking in public settings relates to health and to negative alcohol-related consequences. However, the possibility that increased gender equality may influence alcohol consumption patterns should be accounted for in studies of gender equality and health behaviors of considerable relevance to setting public health priorities in this arena.

## Figures and Tables

**Table 1.** Survey Design Characteristics.

**Country**	**Survey Year**	**Women (n)**	**Men (n)**	**Sampling Frame**	**Survey Mode**
**Argentina**	2003	598	401	Regional: ≈95% of population (Buenos Aires City & Province)	Face-to-face
**Australia**	2007	1,221	831	Regional (Victoria)	Telephone
**Belize**	2005	1,913	1,721	National	Face-to-face
**Brazil**	2001/2002	387	273	Regional: (Botucatu, Sao Paulo State)	Face-to-face
**Canada**	2004	6,904	5,360	National	Telephone
**Costa Rica**	2003	776	381	Regional: ≈50% of population (Greater Metropolitan Area)	Face-to-face
**Denmark**	2003	881	711	National	Telephone
**Iceland**	2001	1,067	931	National: Sampled using Register	Postal/Telephone
**India**	2003	1,215	1,318	Regional: (Karnataka, 5 regions including Bangalore)	Face-to-face
**Isle of Man**	2006	425	366	National	Mixed mode (57.5% F-to-F; 42.5% Tel)
**Japan**	2001	992	993	National	Self-Admin Q
**Kazakhstan**	2002/2003	545	487	Regional (east Kazakhstan)	Face-to-face
**New Zealand**	2007	902	689	National	Postal
**Nicaragua**	2005	1,390	594	Regional: (Bluefields, Esteli, Juigalpa, Leon, & Rivas)	Face-to-face
**Nigeria**	2003	926	1,068	Regional: 2 South, 3 North states & Federal Capital	Face-to-face
**Spain**	2002	716	721	Regional	Face-to-face
**Sri Lanka**	2002	552	543	Near National: 17 of 25 districts	Face-to-face
**Sweden**	2002	954	870	National	Telephone
**Uganda**	2003	743	695	Regional: 1 district in each of 4 regions	Face-to-face
**UK**	2004	863	810	National	Face-to-face
**Uruguay**	2004	624	376	National	Face-to-face
**USA**	2000	3,338	3,057	National: 50 states & Washington DC	Telephone

**Table 2.** Mean Frequencies of Drinking in Public and Private Venues by Country.^a^

**Country/Survey**	**Women**				**Men**			
	**N**	**% Current Drinkers**	**Average Frequency Public Drinking^b^**	**Average Frequency Private Drinking^b^**	**N**	**% Current Drinkers**	**Average Frequency Public Drinking^b^**	**Average Frequency Private Drinking^b^**
Argentina	598	73.7	5.71	41.05	401	91.5	17.64	112.56
Australia	1,221	84.3	22.39	75.58	831	90.0	34.57	118.74
Belize	1,913	20.1	3.72	5.95	1,721	52.9	19.36	20.04
Brazil	387	18.9	2.93	9.29	273	39.2	21.02	24.37
Canada	6,904	76.9	17.78	47.36	5,360	83.1	20.93	75.34
Costa Rica	776	45.4	7.73	7.83	381	69.8	23.47	14.09
Denmark	881	93.8	18.10	67.46	711	96.8	31.67	99.56
Iceland	1,067	86.1	5.69	22.63	931	87.3	23.83	36.40
India	1,215	3.0	.14	1.80	1,318	36.9	37.07	8.49
Isle of Man	425	88.0	27.74	78.33	366	95.4	62.04	105.59
Japan	992	78.7	10.40	69.49	993	92.0	29.55	169.89
Kazakhstan	545	66.6	.92	9.21	487	77.2	2.96	20.80
New Zealand	902	90.4	25.80	96.89	689	90.1	40.32	101.52
Nicaragua	1,390	10.7	3.11	2.86	594	44.1	19.05	19.18
Nigeria	926	20.8	11.34	13.29	1,068	40.8	32.95	28.29
Spain	716	51.1	25.63	49.45	721	72.8	87.64	123.90
Sri Lanka	552	5.8	.07	.34	543	56.5	9.68	14.19
Sweden	954	64.9	12.58	41.21	870	78.9	20.48	60.32
Uganda	743	39.6	16.44	17.99	695	54.2	60.78	22.49
UK	863	84.2	31.93	72.79	810	91.5	69.60	95.41
Uruguay	624	60.3	4.98	27.47	376	81.1	14.00	63.13
USA	3,338	60.4	10.43	17.27	3,057	68.8	23.53	34.55

**All Countries**	27,932	59.0	13.17	35.02	23,196	72.6	31.53	61.02

a Note Ns are unweighted;

b Means are weighted and include those indicating no drinking in venues.

**Table 3.** Pearson Correlation Coefficients between Country-Level Variables.

**Country-Level Variables**	**Gross Domestic Product**	**Gender Empowerment Measure**	**Economic Participation and Opportunity**	**Educational Attainment**	**Political Participation**	**Reproductive Autonomy**	**Context of Violence Against Women**
**GDP**	1	0.69	0.36	0.24	0.51	0.73	−0.69
**GEM**	--	1	0.67	0.53	0.82	0.76	−0.59
**EP&O**	--	--	1	0.20	0.64	0.48	−0.32
**EA**	--	--	--	1	0.36	0.47	−0.34
**PP**	--	--	--	--	1	0.84	−0.66
**RA**	--	--	--	--	--	1	−0.86
**CVAW**	--	--	--	--	--	--	1

**Table 4.** Coefficients for the 2-Level Model Predicting Frequency of Drinking in Public & Private.

	Public Venues	Private Venues

**Model 1:***Base Model controlling for Age and Marital Status (no Country Level Predictors)*^a^		
Intercept	1.071 (0.181)***	1.694 (0.235)***
Age	−0.017 (0.003)***	−0.003 (0.003)
Marital Status	−0.174 (0.041)***	0.165 (0.041)***
Gender	0.697 (0.062)***	0.737 (0.083)***

**Model 2:***Country–Level Coefficients Predicting the Country–level Gender Coefficients (Each Included in Separate Models)*^a^, ^b^, ^c^		
*Economic Status :*		

Gross Domestic Product	−0.161 (0.032)***	−0.065 (0.055)
*Gender Equality:*		
Gender Empowerment Measure	−0.153 (0.042)***	−0.066 (0.072)
Economic Participation & Opportunity	−0.210 (0.026)***	−0.136 (0.063)
Educational Attainment	−0.114 (0.043)**	0.071 (0.032)*
Political Participation	−0.019 (0.075)	−0.056 (0.074)
Reproductive Autonomy Factor	−0.144 (0.041)***	−0.003 (0.003)
Violence Against Women Factor	0.175 (0.055)***	0.008 (0.061)

**Model 3:***Country–Level Coefficients Predicting the Country–Level Gender Coefficients Included Simultaneously^a^,^b^,^c^*		
Gross Domestic Product	0.042 (0.071)	−0.142 (0.071)*
Economic Participation & Opportunity	−0.221 (0.062)**	––
Educational Attainment	––	−0.075 (0.772)

a For Model 1 (for private and public venues), each of the separate models in Model 2, and Model 3 the estimates of the variances of the random effects for both the random intercept and gender slope coefficients were found to be significant at the .001 level indicating significant variation across countries.

b Models 2 and 3 control for individual level age and marital status.

c Coefficients shown for Models 2 and 3 are only for the gender coefficient (difference in log frequency of drinking between men and women) but were also included as predictors of the random intercept.
